# Patient and Health Care Provider Experiences With Suicide-Related Tele–Mental Health Evaluations in the Emergency Department: Multiphase Qualitative Study

**DOI:** 10.2196/72541

**Published:** 2025-06-26

**Authors:** Aishwarya Khanna, Celine Larkin, Rachel Davis-Martin, Ivy Khevali Micklus, Ana Vallejo Sefair, Ancella Roy, Christian Guy Klaucke, Martin A Reznek, Edwin D Boudreaux

**Affiliations:** 1 Department of Emergency Medicine University of Massachusetts Chan Medical School Worcester, MA United States; 2 Department of Family Medicine and Community Health University of Massachusetts Chan Medical School Worcester, MA United States; 3 Department of Psychiatry Boston University Chobanian & Avedisian School of Medicine Boston United States; 4 Department of Psychiatry University of Massachusetts Chan Medical School Worcester, MA United States; 5 Department of Population and Quantitative Health Sciences University of Massachusetts Chan Medical School Worcester, MA United States

**Keywords:** suicide prevention, emergency department, telehealth, patient perspectives, implementation, provider perspectives

## Abstract

**Background:**

Suicide is one of the most pressing public health issues in the United States, inflicting a devastating toll on families, communities, and society. Individuals with suicide risk often visit emergency departments (EDs), but the setting has chronic shortages in psychiatric care staffing, which results in gaps in best practices, prolonged length of stay for patients, and unnecessary inpatient admissions. To improve behavioral health care and suicide prevention practices, we implemented telehealth-based mental health evaluations with enhanced suicide care at 2 EDs in Massachusetts. Little is known about patient experiences and perceptions toward the appropriateness of telehealth for emergency mental health evaluations in the context of suicide prevention.

**Objective:**

The goal of our qualitative study was to understand patient and health care provider experiences with the Telehealth to Improve Prevention of Suicide (TIPS) program and to gain insight into aspects of the implementation process.

**Methods:**

We conducted 25 semistructured qualitative interviews with 10 patients who received a tele–mental health evaluation and 12 clinicians, including behavioral health and ED providers, whose clinical workflows included the new telehealth implementation. We used methods for rapid qualitative analysis and were guided by key implementation of a priori domains outlined in the Practical, Robust Implementation and Sustainability Model framework.

**Results:**

Patients and health care providers reported their perceptions of the patient care experiences and recommendations related to implementation. Patients’ perspectives were highly varied, with several factors and priorities contributing to their views on tele–mental health in this setting. Overall, patients valued transparency and informed decision-making, which extended to having the option to choose between an in-person or telehealth evaluation. Health care providers generally felt that in-person evaluations were preferable; however, given the long wait times and staffing concerns, telehealth evaluations offered a strong alternative. Both patients and health care providers reported several recommendations for future implementation efforts, including increased support and information, communication throughout the process, and improving overall psychiatric care in the ED.

**Conclusions:**

Given current shortages in behavioral health care, emergency tele–mental health evaluations could provide an opportunity to reduce wait times and support the delivery of best practice suicide-related care. However, their implementation has the potential to exacerbate existing issues related to patient autonomy, therapeutic alliance, and care transitions. Our study contributes to filling a gap in knowledge related to patient and health care provider experiences of this telehealth service and describes factors that impact implementation, which may inform future care advances by clinicians and administrators.

## Introduction

### Background

Suicide is one of the most pressing public health problems in the United States, and it takes a devastating personal toll on families and communities across the country. In 2022, suicide was the 11th leading cause of death in the United States [1], and suicide rates have continued to rise through at least 2023 [2]. In fact, suicide rates in 2023 were reported as the highest they have been since 1941, indicating an urgent need for prevention efforts [2].

Approximately 1 in 100 emergency department (ED) encounters are characterized by suicidal ideation or behavior as the primary reason for the encounter [3-5] and 3% to 8% of ED patients, regardless of reason for ED evaluation, endorse suicidal ideation or recent behavior when asked through universal screening processes [6-8]. Therefore, the ED stands to be a valuable setting for suicide prevention initiatives. However, implementing suicide prevention practices in the ED is challenging due to several barriers [6,9-11]. First, severe shortages [12] of dedicated, on-site behavioral health specialists [10,13,14] can lead to potentially unnecessary forced transfers, conservative risk stratification, unnecessary psychiatric inpatient admissions, prolonged patient length of stay in the ED, and ED boarding (the practice of holding admitted patients in the ED). In addition, staffing shortages can lead to missed opportunities for evaluations and interventions [12,15,16]. Furthermore, even when behavioral health services in the ED are accessible, current practices can be of suboptimal quality; numerous studies demonstrate poor adherence to suicide-related best practices, including inadequate risk assessment, safety planning, lethal means counseling, and linkage to outpatient behavioral health [17-20].

Telehealth evaluations present an opportunity to mitigate current limitations in access and quality of behavioral health services in the ED. Telehealth approaches in general, including those addressing both medical and psychiatric needs, appear to be increasingly accepted by EDs and health care providers across the nation [11,21-23]. The National Institutes of Health–funded Telehealth to Improve Prevention of Suicide (TIPS) program combined best practice standards in both telehealth videoconferencing service delivery [24] and suicide prevention for implementation in ED settings. The program was designed for translation to other health systems.

In ED settings, studies on tele–mental health have demonstrated benefits to patients and the overall system in reduced time to be seen, disposition, and transfer avoidance [22]. Using telehealth in ED settings to connect patients with clinicians focused on suicide prevention and safety planning is also an emerging area of research and implementation [25]. However, previous literature has not explored patients’ lived experiences and stakeholders’ perspectives of the appropriateness of this modality for suicide prevention in emergency settings. Qualitative methods can address this gap in the literature by describing a range of experiences, providing richer and more granular descriptions of experiences, and enhancing previously established quantitative data [26,27]. Furthermore, qualitative methods are critical for studying implementation and can support evaluation of an intervention [28], providing crucial information for transferability in other settings, particularly for mental health services [29].

### Objectives

The goal of this qualitative study was to ascertain patient and health care provider perceptions of a telehealth program to improve suicide prevention in EDs and its implementation process. We sought to understand current gaps in knowledge related to patient and health care provider experiences with emergency tele–mental health evaluations while also informing recommendations for implementation that may improve suicide prevention efforts in EDs across the nation.

## Methods

### Study Design

This study was implemented at 2 separate EDs in a medium-sized, urban-based academic health care system in Massachusetts. The implementation phase of the study lasted 24 months. Implementation began at one site in July 2021 and at the second site in October 2021. TIPS combined the current best practice standards in both telehealth service delivery [24] and suicide prevention, and it was designed for translation to other health systems. TIPS used a secure or HIPAA (Health Insurance Portability and Accountability Act)–compliant televideo platform for telehealth service delivery but had a telephone option available as a backup if needed. In addition to implementation of emergency tele–mental health evaluations, our team provided enhanced training and feedback for the evaluators focusing on three best practices: (1) suicide risk assessment using the Columbia-Suicide Severity Rating Scale, risk assessment version [30,31]; (2) collaborative safety planning using the Stanley-Brown Safety Planning Intervention [9,32,33]; and (3) care transitions using National Action Alliance for Suicide Prevention Best Practices in Care Transitions for Individuals with Suicide Risk [34].

Semistructured qualitative interviews were conducted with patients who had undergone a mental health evaluation in a TIPS site ED, as well as with clinicians providing tele–mental health services at a TIPS site. Health care provider interviews were completed before TIPS implementation and after the implementation of TIPS. This was done to understand evolving facilitators of and barriers to implementation and understand perceptions around the TIPS program. Preimplementation interviews were also used to inform implementation strategies. All patient interviews were completed after implementation to understand patient experiences with TIPS and perceptions around tele–mental health emergency evaluations.

### Ethical Considerations

The University of Massachusetts Chan Medical School Institutional Review Board (docket number H00021007) approved the TIPS study and the study procedures described in subsequent sections. All participants provided verbal informed consent to participate in this study. Health care providers were informed about the purpose of the study and the voluntary and confidential nature of participation. No compensation was provided for either patient or health care provider participation.

### Participants and Recruitment

Participants included patients and health care providers (ie, attendings, ED administrators, nurses, and behavioral health providers). Potential patient participants were identified in the electronic health record (EHR) as having undergone an emergency mental health evaluation between December 2023 and June 2024. In this timeframe, 122 patients were identified in the EHR as having undergone an emergency mental health evaluation, and of these, 74 (60.6%) were considered eligible. Eligible patients were those who had a valid phone number and home address recorded in the EHR; English speaking; over the age of 18 years; not presently incarcerated or pregnant; and those who had no history of memory impairment (ie, dementia or paranoia at the time of presentation). These patients were mailed a consent factsheet and letter inviting them to participate in the study. Patients were identified and screened for eligibility by a research assistant (IKM) and the same staff member mailed the factsheet and letter. Patients’ individual decisions on whether to participate were not shared with their treatment team. Two weeks after mailing the factsheet and letter, one study member (AK) called the patients and invited them to participate over the phone, with up to a total of 3 attempts to contact each patient. Once a patient was reached and if they were willing to participate, they were asked to verbally consent before participating in the interview. No prior relationship existed with patient participants, and patients were briefed on the interviewer’s (AK) purpose and interests in the study. We initially planned to conduct 10 patient interviews per implementation phase (10 before the implementation, 10 at 6 months, 10 at 12 months, and 10 after the implementation), with plans to continue until thematic saturation was achieved. However, given that we would have needed to approach patients in the ED in real time to meet this goal and ethical considerations of approaching patients who may be in crisis, we opted to complete interviews after the implementation only following the aforementioned protocol. Thematic saturation was achieved with our 10 interviews, and we opted to stop recruiting additional patients.

Potential health care provider participants were those who identified as being involved in the TIPS implementation or service provision and were invited by email, with a study factsheet, to participate in a qualitative interview by a researcher and a junior faculty member CL. Health care providers’ individual decision on whether to participate was not shared with the implementation team or leadership. We aimed to recruit 10 health care providers across both rounds, with plans to recruit additional ones as needed to achieve thematic saturation. Health care providers were asked to verbally consent to participate before participating in the interview.

### Data Collection

Patient data collection was completed by one female study staff member (AK) who was trained and experienced in qualitative study design. Health care provider data collection was completed by another female study staff member (CL) who was also trained and experienced in qualitative study design.

Semistructured qualitative interviews followed an interview guide (Multimedia Appendix 1) and did not last more than an hour. The topic guide was based on the Practical, Robust Implementation and Sustainability Model (PRISM) implementation framework. The PRISM framework was chosen to guide our interviews as it is developed to provide research teams with pragmatic insights that guide implementation and help make interventions clinically translatable [35,36]. Domains under the PRISM framework include multilevel perspectives on the intervention, multilevel characteristics of recipients or stakeholders, the external environment, and the implementation and sustainability infrastructure. Our guide explored participants’ perceptions across all PRISM domains: the TIPS program overall; patients’ and health care providers’ perceived characteristics; characteristics of the ED; external context that may be relevant to implementation; and thoughts on the implementation process, sustainability, and recommendations. Health care provider interviews were completed via Zoom (Zoom Communications, Inc) [37], audio recorded, and transcribed verbatim by a study team member (AK). Patient interviews were completed over the phone, audio recorded, and transcribed verbatim by a study team member (AK). Data collection was completed in a home or workplace setting using a secure phone designated only for study purposes. Data collection for patients after the implementation was completed between January 2024 and July 2024. Data collection for health care providers before the implementation took place between May 2021 and June 2021 and after the implementation took place between March 2023 and April 2023, following the TIPS program’s launch date on July 12, 2021.

### Data Analysis

We used the same approach to data analysis for both health care provider and patient interviews. We used a rapid approach [38] to a directed content analysis [28] to understand participants’ views of the TIPS program and their experiences with implementation. Two study team members (CL and AK) summarized individual interviews using a structured template that followed a priori domains outlined by the PRISM framework and were included in our interview guide. Domains determined in advance of data analysis were derived from the PRISM framework as outlined earlier.

Two random transcripts per participant group were independently coded by study team members (CL and AK), who then met to compare consistency in the application of the template and to develop consensus in our review. The remaining transcripts were then divided and coded independently by CL and AK and discussed through regular consensus-building discussions. After primary coding, a secondary coding check was also completed on each transcript, whereby the second study team member reviewed the transcript to capture additional content or to check agreement.

Summaries were then consolidated into a single matrix document, and using constant comparison, summaries were synthesized within and across domains to guide the understanding of study objectives. The software used to collate and manage the data was Microsoft Excel.

## Results

### Overview

In total, 10 patients and 12 health care providers participated in a total of 25 qualitative interviews via the telephone or videoconferencing software.

Patient interviews were conducted in one round after the TIPS program was implemented via the telephone. Of the 10 patients, 8 (80%) patients identified as White, non-Hispanic, and having an evaluation via videoconferencing-based telehealth. Ages ranged from 18 to 69 years with a median age of 31 years. In total, 6 (60%) patients identified as women and 4 (40%) identified as men.

A total of 12 health care providers participated in interviews via videoconferencing software. Health care provider interviews were conducted in 2 rounds: before implementation and after implementation. A total of 15 interviews were completed across both rounds, with 12 health care providers participating in total (3 participants in the preimplementation round were again interviewed in the postimplementation round). Participants had a range of clinical roles including ED attendings, nurses, and behavioral health providers, and several of them also had administrative or leadership roles. Patient and health care provider demographics are summarized in Table 1.

Consolidation and synthesis of qualitative interviews are presented as domains from the PRISM model, with key messages presented within each domain as relevant. Additional quotes are presented in Textbox 1.

**Table 1:** Participant demographics.

				Participants, n (%)
**Patients (n=10)**				
	**Age range (y)**			
		18-25	3 (30)	
		25-35	2 (20)	
		35-45	3 (30)	
		45-55	1 (10)	
		55-65	0 (10)	
		65-75	1 (10)	
	**Gender**			
		Man	4 (40)	
		Woman	6 (60)	
		Nonbinary	0 (0)	
	**Race**			
		American Indian or Alaska Native	0 (0)	
		Asian	0 (0)	
		Black	0 (0)	
		Native Hawaiian or other Pacific Islander	0 (0)	
		White	10 (100)	
	**Ethnicity**			
		Hispanic or Latino	2 (20)	
		Non-Hispanic or Latino	8 (80)	
	Evaluation via videoconferencing telehealth		8 (80)	
	In-person evaluation		2 (20)	
**Health care providers (n=12)**				
	**Age range (y)**			
		25-35	3 (25)	
		35-45	4 (33)	
		45-55	2 (17)	
		55-65	2 (17)	
		65-75	1 (8)	
	**Gender**			
		Male	7 (58)	
		Female	5 (42)	
		Nonbinary	0 (0)	
	**Clinical role**			
		Nurse	2 (17)	
		Attending	7 (58)	
		Behavioral health	3 (25)	
	Administrative or leadership role		4 (33)	
	**Round of interview (n=15)**			
		Before implementation	6 (40)	
		After implementation	9 (60)

**Representative quotes by the Practical, Robust Implementation, and Sustainability Model domains.**

**Recipient characteristics**

- “Communication is key...[patients should be told] ‘if you [want to] talk in person, you’ll have to wait another eight hours. Is that what you want?’ And give the patient the option of saying ‘no, that’s too long. I’ll do telehealth.’” [Patient 9]
- “I think genuinely everybody wants to do the right thing for the mental health patients...you don’t maybe have the same skill set that you need to work with mental health patients for longer...I think it takes a lot of patience...And sometimes in a busy ER, people just don’t have that kind of patience that it takes to help these people when they’re in crisis...ER nurses aren’t necessarily trained very well to seed out the priority when these other patients aren’t doing well physiologically.” [ED nurse 6]

**Perspectives on the intervention**

- “They sent me into the direction of the [group therapy]...my main stressor was I had just moved up here. I really had no one at that time. It was really hard for me. So when I went to the [group therapy] program, I met a lot of people and that’s brought down stressors a ton.” [Patient 4]
- “My whole point whenever I go in there is, I would say if I was a problem to anyone else or others, I wouldn’t be here looking for help. I’d be out there being a problem.” [Patient 1]
- “Occasionally [we] would have patients here for 3-4 or five days waiting for inpatient placement. We have no place for them to shower...it’s a little outside of our experience in terms of how to manage these patients...We try to get resources like just to keep the patients engaged...books, magazines, iPads, something to help them pass the time...And then also just in terms of trying to provide some sort of ongoing management...it’s all psychiatric. There’s no medical issue. They’re evaluated by Behavioral Health...They need to go inpatient. And then 72 hours go by, and they haven’t been placed yet. So, they’re just sitting here, right. And you know, they get agitated, and we give them some antipsychotic medications. So, they sleep for a few hours, but [we’re] not really treating...we’re going to have boarders, [and] we’ll need to have some sort of system to manage them better because we weren’t really doing them a service at all.” [ED attending 7]
- “It’s possible to do it if you put the time and effort and empathy in doing it. I think a lot of people, especially who are afraid to go in, like me, because you don’t want to confront whatever is going on with you, Telehealth could be a way to ease a person into building rapport and relationship...you’re still working with them, you’re not working against them, and you’re not denying them that service and they have the option.” [Patient 8]
- “I actually like seeing a person in my presence...There’s just no connection I feel like it’s just somebody on the screen. I feel like that’s really important to feel connected to your care team because mental health is pretty serious and I just feel like telehealth is not what’s best.” [Patient 7]
- “I don’t know if you get the full perspective on [telehealth]...especially with behavioral health, there’s a lot of nuance that you may not pick up on...general hygiene, appearance. You can see basically neck up, but you’re not sure about the rest of their appearance.” [ED attending 3]

**Fit between recipient characteristics and intervention perspectives**

- “Everybody else treated me like an idiot basically. They didn’t talk to me. They would talk about me and I could hear them. I was referred to as a drunk. I was there for mental health and I was there for addiction services, but I was not treated like a human being. I’m very pissed off about that. I’ve been a social worker for over 25 years—first [time] finally admitting that I have a problem and I’m asking for help, and I get treated like shit.” [Patient 8]
- “It almost seemed like they were ticking boxes, rather than actually seeing what was wrong with me. Now, we’ve moved past ‘I’m not thinking of hurting myself or others’. You [can] actually talk to me as a person. Whatever, they didn’t end up sectioning me. So that made me happy because I don’t need to be incarcerated at a hospital because I’m depressed. That’s not going to help anything.” [Patient 1]
- “I remember answering some of her questions, but I didn’t want to be discussing this. So, I wasn’t sharing information. I wasn’t giving them everything that they were looking for.” [Patient 2]
- “There was no [communication] until they came in and told me I was leaving to go to a [psychiatric hospital]. I said what? What do you mean? I think that telehealth needs follow up with the patient and talk about the decision that they’re making instead of just making it. Explain to the patient why we’ve come to this determination. There’s really no communication. Hours and hours and hours and hours go by and nobody talks to you. Nobody tells you what’s going on. I don’t think that’s right. You can’t even do anything. You can’t even leave.” [Patient 9]
- “I don’t have an issue with how everything went with [the clinician]. She seemed great, conversation was fine, she was very professional. I just don’t understand why I was kept there for a week and not helped whatsoever. There [were] no meetings, help, assistance, programs, setting up, nothing. I just sat in a room by myself all day, every day for a week.” [Patient 5]
- “When you do it over the iPad, it’s like, I have another question for you. OK, I have another question for you. There was no follow up. No divulging into any of the questions. Anybody who’s smart enough…if they wanted to leave that hospital and just be discharged, they could have easily told this person whatever they wanted to hear and [been] cleared.” [Patient 8]
- “I mean the conversation I had with my clinician was pretty much my ideal conversation. She was asking me questions and I would answer them...I would put my input and then she would put her input and then we would just come up with a plan together...It was afterwards when what she said did not happen. I didn’t get to talk to [a] psychiatrist even though she said I would. The nurse said I would. And the care that I was supposed to get, I didn’t receive.” [Patient 7]
- “These people don’t even have real beds. They actually lay down on cots for days and days on top of each other. It’s overcrowded. It’s not the most conducive to health and Wellness...Now the fear that [healthcare professionals] have is that if you make it too comfortable, people will want to just hang out there...for the longest time we were giving them only like cold sandwiches. Now we give them warm meals...” [Behavioral health provider 1]

### Recipient Characteristics

Within recipient characteristics, participants’ responses primarily spoke to the needs and values of patients and health care providers but also mentioned patients’ demographics and health needs.

#### Patient Characteristics

Despite a wide variety in patients’ backgrounds and reasons for presentation to the ED, their priorities and values were highly aligned. Several patients described their needs for honesty, confidentiality, understanding, compassion, and lack of stigma on behalf of health care providers, with a desire for self-determination: “I feel like - most patients, we are competent, and we can make decisions for ourselves.” Patients valued being seen as individuals and having their situations evaluated on a case-by-case basis rather than a “blanket policy” being applied to them, with one patient remarking, “I just [want to] be listened to and given all my options.” At a concrete level, patients preferred shorter waiting times and valued staying informed about their care and the mental health evaluation and treatment process. Patients strongly prioritized avoiding involuntary psychiatric treatment and sometimes were concerned that the medical care they needed might be overshadowed by a behavioral health history.

Health care providers had a slightly different perspective. Several health care providers noted that many of these patients were without primary care and often required interpreter services in the ED. They felt that patients with suicide risk tend to be local, younger adults (age 20-50 years), with concurrent substance use and generally with life situations that are highly stressful. Several health care providers made distinctions between patients who have suicidal ideation as the primary reason for presenting to the ED and patients who screen positive for suicidality on routine screening, with the former having a more extensive history and being more open to help. The general perception from health care providers was that patients likely would have a positive care experience with telehealth evaluations but could have a relatively more positive experience with in-person evaluations.

#### Health Care Provider Characteristics

In relation to their own motivations and needs, ED providers wanted “to provide care for the patient, ultimately” and described a good day as providing “good care to an appropriate amount of patients, [feeling] appreciated, and [making] some meaningful differences in a person’s life.” Having adequate infrastructure and resources, such as beds, proper machine functioning, and testing resources, were important to avoid providing “marginal care to a large amount of patients and [feeling] like you may have missed something.”

### Perspectives on the Intervention

#### Overview

While we were initially mostly interested in patients’ perspectives of tele–mental health evaluations, the interviews provided a lot of information about patients’ experiences of behavioral health care in the ED more broadly. This helped us to understand the context in which tele–mental health evaluations were being offered, and how they might contrast with more traditional behavioral health care in the ED. Therefore, within this domain, we present first on general perspectives on behavioral health care in the ED, then on behavioral health evaluation in the ED, and finally on telehealth evaluation in the ED.

Generally, patients viewed the ED as a starting place to seek psychosocial help, especially if they were not previously connected with resources such as therapists or psychiatrists. The typical experience tended to be more often negative. Patients described long wait times before being seen by a behavioral health provider; wait times ranged between several hours to up to a day and a half. One patient also described paradoxically experiencing a delay in receiving medical treatments for alcohol use disorder and alcohol-induced pancreatitis until he was first seen and cleared by behavioral health. Patients also described instances of stigma for seeking help for behavioral health–related concerns.

Several patients commented on poor communication that led to them feeling ignored or misinformed. A few patients shared that they did not receive services that were promised or agreed upon (eg, psychiatrist coming, medications being changed, and neuropsychiatric evaluation not being received): “they say things are going to happen and they don’t happen.” Other patients felt lied to or kept in the dark about what was happening with one patient stating, “every single person lied to me about how long I was going to be there, so I wouldn’t lose it.” Another patient shared that a physician did tests that did not make sense and did not explain how it would help in care. One patient recalled being administered an intramuscular antipsychotic that she did not consent to during her stay, without being told why it was necessary.

Patients also had negative experiences related to certain policies and procedures, with involuntary psychiatric holds being most frequently discussed. One patient felt that health care providers were complicit in isolating and causing harm to behavioral health patients, stating, “they take you away from all of your supports and they put you in a place where you have nothing.” Some patients reported a level of distrust in health care providers’ motivations, with one suggesting a health care provider kept them on involuntary psychiatric hold due to lack of insurance.

#### Perspectives on Mental Health Evaluations in the ED

Patients’ experiences with mental health evaluations were mixed. Patients described the evaluation as being short, typically lasting 5 to 30 minutes, and consisting of several questions around the patient’s medical history, medications, and what led them to the ED*.* Patients stated that they were asked protocolized questions during their mental health evaluations and perceived health care providers as engaging in conservative risk management. This resulted in some patients feeling treated “like cattle,” while also feeling clinicians “did what they could with what they had. It’s just their hands are tied in a lot of situations.” Positive experiences were described as the evaluation resulting in some connection to resources or outpatient behavioral health support. Some patients also described positive experiences as those that did not result in an involuntary psychiatric hold or hospitalization after evaluation.

#### Perspectives on Tele–Mental Health

Patients reported a range of previous experiences with tele–mental health outside of the ED, with several patients describing barriers around insurance coverage and long wait times for accessing outpatient tele–mental health. The general perception from health care providers on telehealth evaluations in the ED was that in-person evaluations would be a better option preferred by patients but that, given the barriers, long wait times, and need for transfer, telehealth was the next best option.

**Figure 1:**
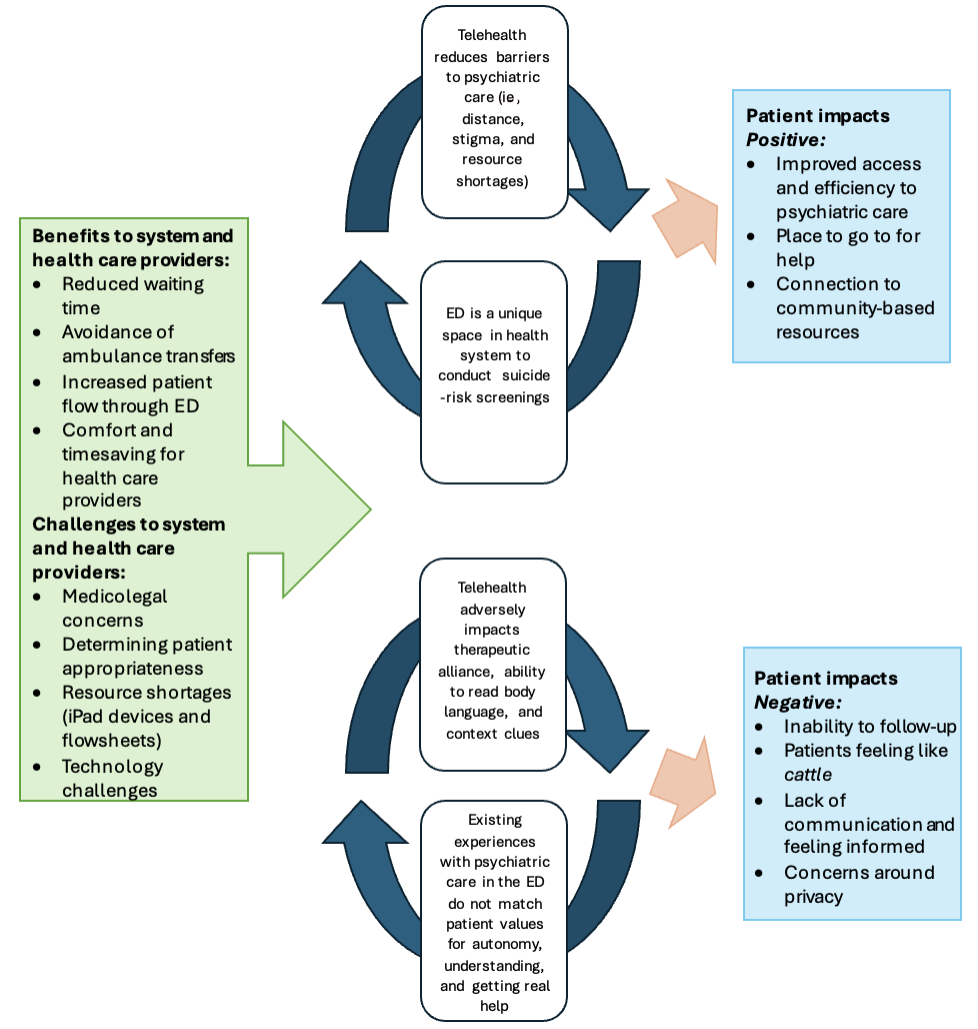
Summary model of telehealth's impact on psychiatric care in the emergency department (ED).

Most participants felt that deciding between telehealth versus in-person evaluation should be achieved through shared decision-making, reflecting the wish for informed choice mentioned earlier. Patients who preferred telehealth cited ease of access, increased comfort, decreased wait time, and avoidance of transfers as reasons for their preference. Those who preferred in-person cited interpersonal factors such as increased rapport, feeling more understood in person, ability to obtain collateral, and judging body language and maintaining eye contact. The themes in this section and the previous are depicted in our summary framework for telehealth’s impact on psychiatric care in the ED (Figure 1). Patients and health care providers perceived the main advantages of telehealth to be the potential for reduced wait time for evaluation, increased accessibility, and avoidance of ambulance transfer, thereby reducing costs and time in the ED.

From a health care provider perspective, telehealth evaluations were broadly seen as acceptable to patients, if not as good as an in-person evaluation. Telehealth evaluations were a way of reducing dissatisfaction and agitation because patients were seen more quickly with less moving around, which was perceived to “[mean] so much to patients.” Telehealth was perceived as particularly helpful in cases where a patient had mobility issues or interpreter needs.

About half of the patients also agreed that despite technical difficulties, telehealth was preferable to prolonged wait times; it was described by one participant as “way better than having to wait a day and a half” as was experienced previously in an in-person evaluation. Several patients preferred telehealth in an ED because it was perceived to reduce wait times compared to in-person evaluations. Several participants commented on improved accessibility with telehealth. One patient perceived telehealth to feel like an easier way to engage with mental health supports than in-person interventions, particularly for those who are resistant or scared to seek mental health care.

Several health care providers also felt that telehealth would lead to a better use of limited health system resources, such as beds and ambulances, with some clinicians noting that telehealth at one hospital meant being able to take some of the burden off the sole hospital with embedded emergency mental health services, carrying “benefits to the system as a whole.” Advantages to the behavioral health clinicians conducting the evaluations included being able to see the health record while talking to the patient. They also cited the advantage of not having to travel to a distant site, thus saving them time, especially in bad weather. One respondent mentioned that if behavioral health clinicians were now allowed to work from home, it could “reduce burnout and attrition.” One behavioral health clinician described it as a “good, quick, easy way” to see patients. Patients also noted this advantage of telehealth for clinician well-being and perceived that if a clinician is less stressed while in their own environment and setting via telehealth, they might be able to do their jobs more effectively. Patients shared that TIPS would be feasible only if all parties felt comfortable discussing sensitive topics in their respective environments.

### Fit Between Recipient Characteristics and Intervention Perspectives

The PRISM model depicts a bidirectional relationship between recipient characteristics and intervention perspectives. In this case, there were discrepancies between recipients’ needs and preferences on the one hand and what care was being offered on the other hand. We present this domain more generally in relation to behavioral health care in the ED and then with a focus on tele–mental health in the ED.

#### Behavioral Health Care in the ED Not Meeting Patient Needs

Relating to the desire to make informed choices about their own care, most patients expressed a wish to be more informed about the evaluation process and to have increased autonomy over the situation. Several patients described feeling unaware about what was happening and what the potential outcomes of the evaluation would be. A few patients were surprised to be undergoing a mental health evaluation without first being asked about possible physical symptoms that may be contributing to their presentation. Finally, several patients perceived a lack of closure or explanation around the next steps after their evaluation or admission based on involuntary psychiatric hold.

Several patients shared that they felt that the evaluation questions were repetitive and checkbox in nature, and that the clinician was more concerned with getting through the questions rather than assessing the patient themselves. Patients perceived the questions as restrictive and felt that they did not allow them to engage as individuals and went against their need to feel human and heard. Some patients also felt that the nature of the questions precluded the clinician from delving deeper into the reason for the patient’s presentation, which may adversely impact care, and could make it easy for the patient to lie to avoid admission. One patient perceived that he was “penalized for answering with honesty” and “traumatized” because of a 3-day inpatient psychiatric hold. Some patients felt believed and trusted while others did not. Those who did not feel believed by the clinician stated this was because the evaluation itself was short, and the clinician did not know the patient or their broader life, or one patient feeling that collateral accounts were prioritized over their own story: “They don’t know me from a hole in the wall.” One patient, who received an in-person evaluation, was unhappy about being told she was undergoing an evaluation, which impacted rapport from the start.

Finally, although patients expressed a desire to obtain effective help and support, reactions to the evaluation varied across patients. The few patients who were satisfied with the outcome of their evaluation felt that the clinician “did everything [they] were supposed to” in terms of connecting patients with resources that improved their mental health shortly (ie, within ED visit) after evaluation and not placing them on an involuntary psychiatric hold. Most patients were not happy with what resulted from their evaluation. Factors that contributed to dissatisfaction included placement on an involuntary psychiatric hold, feeling forgotten about in the ED, no connection to resources, and a lack of communication from health care providers. Participants who were placed on an involuntary psychiatric hold shared that the experience was traumatizing, with some stating that their “mental health is worse” as a result. While admitted, patients shared that they were not given any information about how long they would be there for, any additional resources, or a long-term plan for care.

Patients also shared that not receiving the outpatient follow-up care that was discussed during the evaluation was also a point of frustration. One patient shared that the evaluation itself was ideal because she felt heard and agreed to the plan that was formed with shared decision-making, but there was no follow-through after leaving the ED. Several patients were told that they would be connected to services (such as counseling, peer help groups, therapists, and psychiatrists), but never heard anything about how this would happen, and had to wait several days to hear more, or were never connected.

#### Tele–Mental Health in the ED and Patient Needs

Regarding patient autonomy and desire for informed choice, patients often were not given an option of in-person or telehealth evaluation nor additional information about what each of those options would mean for the patient and their care (ie, need for a hospital transfer). Telehealth also adversely affected patients’ ability to ask questions afterward, with one patient stating: “I finished talking to [the clinician] ... she leaves and then I can’t get any answers from anyone about anything.” Some patients also shared concerns around preserving privacy during a tele–mental health evaluation.

Regarding feeling humanized and heard, both participant groups commented on the loss of certain in-person components, which was perceived to adversely impact patient–health care provider relationships and the quality of evaluation. For example, several health care providers felt that telehealth evaluations are less personal due to a perception that patients may feel less willing to open up over telehealth versus an in-person intervention. Some patients also perceived that a telehealth evaluation may not be taken as seriously by health care providers as an in-person evaluation. This was perceived by patients as the clinician rushing through the evaluation or patients “fudging” responses. Most patients also agreed that not having the health care provider in person adversely impacts the feeling of connection.

Patients described a few lags with the technology, with some patients stating that the clinician on the other end of the line may also be experiencing difficulty connecting leading to confusion and wait time: “for 15-20 mins there was no one at the other end of the line,” had to wait, “it took so long and I was just feeling so miserable [while] detoxing.” Many health care providers felt that having a limited view of a patient’s nonverbal cues, personal hygiene, and general appearance, as is the case with videoconferencing telehealth, could affect the quality of the evaluation.

Patients agreed and stated that being able to read a health care provider’s body language is also useful to them during evaluations. One behavioral health clinician also stated that sometimes patients are very somnolent and not being there in person to physically nudge them awake impacts care. In contrast, several patients felt like they could speak with the clinician freely via telehealth and that the conversation felt very natural. One patient also shared that she was told she would receive a copy of her suicide safety plan but never did.

### Implementation Infrastructure

Patient recommendations for implementation were mostly around increased supports for patients. This came in the form of more information and practical support from ED staff. Patients stated that they felt unprepared for a mental health evaluation and the overall process, and some stated that they did not know the evaluation would take place over telehealth until it happened. Specific recommendations on preparation and information included the following: creating a breakdown of the process and making this available to patients in the form of an information sheet; offering patients an opportunity to write things down before evaluation and giving them an idea of what questions may be asked; and having someone in person explaining the process of a mental health evaluation. Patients wanted better communication around why the patient would be undergoing a mental health evaluation, the option of telehealth versus in-person evaluation, follow-up after evaluation to close the loop and explain the plan to the patient, and more support and engagement from ED staff. Patients also recommended additional training for behavioral health providers on navigating the technology and troubleshooting issues, as these were mostly handled by ED staff. Finally, one patient stated that ideally a mental health evaluation would include some long-term planning or thinking around outpatient mental health needs: “I suppose after getting past the necessary questions that they have to [and] asking, ‘What do you need to stop coming [to the ED] so often?’”

Health care provider recommendations were mostly around the preparation phase of implementation. Multiple participants felt that the preparation could have been more intensive and that it might have been helpful to introduce TIPS as 2 different changes: first, implementing in-person mental health evaluations at the hospital, as one TIPS ED was not previously offering the service in any form, and second, doing those evaluations as telehealth. They spoke of the need for training and awareness for all roles, from secretaries to physicians, for example, by attending huddles and doing robust hands-on preparation with clinicians, demonstrating the telehealth cart, and having a team representative available in the ED to troubleshoot. One participant mentioned the importance of leadership buy-in, and 2 participants mentioned the need for improved communication and understanding between ED and behavioral health providers. The additional need for role clarity within the workflows was mentioned including clear expectation setting on turnaround times. Health care providers cited the need for additional physical resources, such as more iPad devices, an EHR-based flow sheet, and a patient leaflet. One participant also mentioned that it would have been helpful to have a backup plan for downtimes, and another wished to offer patients the choice of an in-person evaluation, while avoiding a transfer.

## Discussion

### Principal Findings

This study used the PRISM implementation framework to explore patient perceptions and experiences with mental health telehealth evaluations in the ED and health care provider experiences with implementation.

Our study described patients’ values for care in feeling humanized and heard, autonomy, and getting real help. Health care providers valued ultimately serving patients and having adequate resources (ie, infrastructure and time) to do so. Benefits of the TIPS program were described as emergency telehealth evaluations being a place for patients in crisis to go to for help, improved access to behavioral health services (particularly for those who may be apprehensive to seek care), and improved efficiency of the initial behavioral health assessment. Health care providers also viewed the ED as an appropriate place to detect and intervene on suicide risk, given how many patients at risk for suicide present to the ED. Health care providers also acknowledged that performing suicide risk assessments by telehealth does increase workload for ED staff and an initial learning curve is to be expected, but investing in training and defining roles in the preparatory phase may help with this. Patients also endorsed that proper clinician training is needed for approaching a behavioral health evaluation via telehealth versus in-person interventions, as context clues around the patient get missed over video, and it is perceived to be more challenging to build rapport.

Finally, we found that patient experiences and how they aligned with their values for care greatly varied. While some patients described good experiences of undergoing mental health evaluations in the ED, others described several factors contributing to suboptimal scenarios, having to do with the ED itself or the outcomes of their evaluation. Across both patients and health care providers, there was a consensus that patients should be given a choice about how they would like to undergo a mental health evaluation in the ED, after being informed of all procedural details. It was also found that little information is given to patients about the mental health evaluation process, legal implications, and potential outcomes of evaluation, contributing to several unmet needs and traumatic experiences for patients facing behavioral health concerns.

### Comparison With Prior Work

Telehealth approaches to behavioral health and suicide prevention, more specifically in ED settings, is an emerging area of research. Studies have demonstrated that emergency tele–mental health fills a critical gap in access to psychiatric care, and, in some communities, is the only available form of emergency psychiatric services [39]. Studies have also demonstrated decreased wait time to be seen by a mental health provider [40,41], reduced ED revisit rates, connection to care in the community and reduced inpatient hospitalization, and reduced follow-up encounters involving self-harm diagnosis [41]. The findings of previous studies in this area echo the perceived benefits that were generally brought up around telehealth in our study

However, other studies have also demonstrated that tele–mental health use in ED settings can lead to increased ED length of stay and does not decrease the likelihood of inpatient hospitalization [40,42]. It has also been suggested that telehealth may only be appropriate for certain diagnoses such as depression or anxiety management [40]. For suicide-related care, specifically, it has been shown that ED length of stay is longer than that for patients receiving telehealth care for mood or substance-related disorders [40]. Our study adds to this body of literature by focusing on suicide-related presentations, illustrating what a patient may experience while undergoing risk assessment and safety planning through telehealth in ED settings, and exploring perceptions around appropriateness of the intervention. Furthermore, patients and health care providers shared what the experience is like to be waiting in the ED for next steps in care and how that often does not align with these values.

Studies have also explored factors related to the implementation of tele–mental health approaches in ED settings and reported that increasing patient needs, availability of telehealth resources, and health care provider collaboration are facilitators to tele–mental health adoption. Barriers that have previously been explored include health care providers’ lack of clarity around workflow [43], which mirrors our findings around health care providers’ experiences with implementation. Our study expands on implementation determinants by exploring specific recommendations around what would be helpful for health care providers to be most prepared with the changes.

Furthermore, the context of the ED—particularly for delivering psychiatric and related care—is well-described in a recent qualitative study by Isbell et al [44], which describes the first grounded model of challenges and care dynamics associated with psychiatric care in the ED. This study describes barriers that exist within the ED and the larger health care system that, in combination, contribute to negative experiences felt by patients seeking psychiatric care, typically around feeling stigmatized and decreased quality of psychiatric care delivered in the ED [44]. The findings of this study largely mirror the experiences of patients in our study. This indicates that interventions for emergency tele–mental health must strongly consider the context of the setting in which it is being implemented and aim intervention design around cultural transformation of health care provider attitudes toward patients seeking psychiatric care in the ED to improve patient experiences in care.

### Implications for Practice and Research

Our study raises several practical implications for sites seeking to implement similar programs. The findings of our study suggest that telehealth does not necessarily increase patient autonomy in receiving psychiatric care, and feelings of autonomy are attributed to being well-informed, communication, choice of evaluation method, and being aware of what is happening in their care at all points during the ED stay. Despite patient autonomy in psychiatric care being an important aspect of recovery and furthering an individual toward improved functioning [45,46], previous research has demonstrated tension in determining competency for decision-making with individuals in distress or under the influence of substances, resulting in autonomy for behavioral health patients generally being restricted [46,47]. The findings of our study suggest that an overly conservative approach in restricting autonomy has led to secondary harm to patients, when the intent is to identify and treat suicide risk. Programs seeking to implement emergency tele–mental health, or additional behavioral health services in the ED, must consider how to achieve a balance in preserving autonomy in psychiatric care and balancing patient safety to reduce this secondary harm to patients. Approaches that have been shown to preserve autonomy in psychiatric care include both clinical approaches (ie, shared decision-making) and peer support—yet are seldom integrated with care processes in the ED [45].

Our findings highlight several concrete areas where implementation could be improved to preserve patient autonomy. For example, to improve patient communication and feeling well-informed, several patients and health care providers commented on the need for educational material that depicts the overall evaluation process, patient rights, and explains why an evaluation is taking place. Other practical examples in the program workflow include strategies aimed at making patients feel more prepared for the evaluation, for example, giving patients an opportunity to write things down beforehand and an opportunity to ask health care providers questions after the evaluation. These interventions may be strongly suited to being developed through participatory approaches to research and implementation. Participatory research methods, including co-design or cocreation methods, have increasingly been recommended by agencies, such as the World Health Organization, as an approach to be used in suicide prevention across the globe [48,49]. Previous studies have demonstrated that co-design, when used for suicide prevention, is safe for participants [50,51], promotes feelings of empowerment [50,51], can increase engagement and usefulness of interventions by being highly tailored to local context and culture and shifts away from a “one-size-fits-all” approach [52,53]. Co-design methodology may be the only way to successfully honor and incorporate patient experiences in interventions. Furthermore, maintaining a trauma-informed approach and operating from the viewpoint of lived experiences are especially important in suicide prevention, yet this approach is seldom demonstrated in the literature. Future research is needed to study the impact of a co-designed intervention on patient experiences with suicide-related care in the ED and how these interventions impact suicide-related outcomes.

### Strengths and Limitations

This qualitative study aids in filling this knowledge gap and is well suited to characterize how patients perceive and experience a modality that has the potential to bridge gaps in quality of and access to resources related to suicide-preventive care. Furthermore, qualitatively describing health care provider experiences with implementation aids in informing emergency telehealth evaluations at other institutions [28,54]. We used the PRISM implementation framework to guide data collection and analysis [36]. This is strongly suited for our study as we included both patients and health care providers as “recipients” and were interested in the fit between the intervention and the challenging environment of the ED.

Our study has several limitations. First, our patient sample only included housed, insured, English-speaking participants, and patients who were not experiencing paranoia, dementia, or with a disability that may make it challenging to communicate via telephone. These criteria were selected for the feasibility of data collection but made it difficult to assess the impact on health equity and differing perspectives. However, our patient participant pool displayed diversity across ages, occupations, comorbidities, and previous experiences with mental health evaluation. In addition, we initially planned to complete patient interviews across our implementation phases but were limited by ethical considerations regarding approaching this methodology, staffing challenges, and time constraints needed for recruitment. As such, we are unable to see how patient perspectives change over time, but we feel that the most valuable piece of the initial plan was to explore patient perspectives after experiencing the intervention. An additional limitation for the health care provider interviews is that they largely focused on emergency physicians and therefore other clinical roles, such as psychiatrists, nurses, and medical assistants, were not well-represented.

### Conclusions

Telehealth holds the potential to support the delivery of suicide-related care in ED settings. This qualitative study describes patients’ experiences with behavioral health care in the ED and with emergency tele–mental health evaluations. We describe how tele–mental health delivery in the ED may potentially exacerbate existing shortcomings in the ED around patient autonomy, therapeutic alliance, and care transitions. We also report positive experiences with emergency tele–mental health evaluations, which were described as reducing barriers to psychiatric care. Patients provided recommendations around improving information received and communication in the ED. Health care provider recommendations focused on implementing tele–mental health evaluations in the ED, improving workflow, and training for role clarity.

## Data Availability

All data generated or analyzed during this study are included in this published article

## Appendix

Multimedia Appendix 1 Patient interview guide.

## Abbreviations

ED: emergency department
EHR: electronic health record
PRISM: Practical, Robust Implementation and Sustainability Model
TIPS: Telehealth to Improve Prevention of Suicide
