# Supplementary material for: Patient and Health Care Provider Experiences With Suicide-Related Tele–Mental Health Evaluations in the Emergency Department: Multiphase Qualitative Study
Source: JMIR Ment Health. 2025 Jun 26;12:e72541. doi: 10.2196/72541 (PMC12246763; doi:10.2196/72541)
Supplement: Multimedia Appendix 1 [file mental_v12i1e72541_app1.docx]

Patient-facing measures and topic guide

***About 2 weeks ago, you were evaluated in the emergency department at Memorial/Clinton hospital [then you were transferred to the University Campus where you were evaluated by the mental health staff (Mem only)]. I want to ask you about your thoughts on this evaluation process***

**A. Open-ended questions based on PRISM model**

1. *RECIPIENT*

**Can you tell me a little about yourself, and your life outside the hospital?**

Prompts:

General: Who do live with, do you work or go to school, where are you based?

Health: How is your health generally? How is your mental health?

Access: Do you have any conditions that might make it hard to use this service, like hearing or vision difficulty, language problems, dexterity/mobility?

Tech comfort: How comfortable are you with technology, like using a smartphone, tablet, computer?

1. *EXTERNAL ENVIRONMENT*

**Can you tell me a little about your health care outside the hospital?**

Prompts: What kind of providers do you visit in the community, what sort of health services are available to you? What mental health services have you used in the past? Have you faced any barriers in accessing mental health care before? Where can you go if you are not feeling well? What would an ideal healthcare situation look like for you?

1. *INTERVENTION*

**Can you tell me about yo behavioral health evaluation? When and where did that happen?**

**How did you feel about the behavioral health services you received? What was your experience with the clinician like?**

Prompts: How freely were you able to speak to the clinician? How well did the clinician do in making you feel heard? To what extent was the consultation helpful to you? What was most helpful/least helpful? What would an ideal consultation look like? What did you expect to get out of the experience, what did you get out of it?

**How did you feel about the way the consultation was introduced to you?**

Prompts: How did you feel when you were told you would be receiving a consultation? What information did you receive before it started? What would you have like to have known before starting?

1. **Client Satisfaction Questionnaire (CSQ-8)**
2. How would you rate the quality of the service you received?

Excellent  Good  Fair  Poor

1. Did you get the kind of service you wanted?

 Yes, definitely  Yes, generally  No, not really  No, definitely not

1. To what extent has our service met your needs?

 Almost all met  Most met  Only a few met  None met

1. If a friend were in need of similar help, would you recommend our service?

 Yes, definitely  Yes, I think so  No, I do not think so  No, definitely not

1. How satisfied are you with the amount of help you received?

 Very satisfied  Mostly satisfied  Indifferent  Quite dissatisfied

1. Have the services you received helped you to deal more effectively with your problems?

 Yes, a great deal  Yes, somewhat  No, did not help  No, made it worse

1. In an overall sense, how satisfied are you with the service you have received?

 Very satisfied  Mostly satisfied  Indifferent  Quite dissatisfied

1. If you were seeking help again, would you come back to our service?

 Yes, definitely  Yes, I think so  No, I do not think so  No definitely not

**Telehealth only: What was it like for you to do the consultation by telehealth (via the screen)?**

Prompts: Did you have any trouble handling the tablet or earphones? Could you see and hear ok? Were you able to find your way around the program? Did anything go wrong with the technology?

| **C. Acceptability, Appropriateness, Feasibility Measures** | Completely disagree | Disagree | Neither agree nor disagree | Agree | Completely agree |
| --- | --- | --- | --- | --- | --- |
| 1. This service meets my approval. | □ | □ | □ | □ | □ |
| 2. This service is appealing to me. | □ | □ | □ | □ | □ |
| 3. I like this service. | □ | □ | □ | □ | □ |
| 4. I welcome this service. | □ | □ | □ | □ | □ |
| 5. This service seems fitting. | □ | □ | □ | □ | □ |
| 6. This service seems suitable. | □ | □ | □ | □ | □ |
| 7. This service seems applicable. | □ | □ | □ | □ | □ |
| 8. This service seems like a good match. | □ | □ | □ | □ | □ |

| **D. Usability of telehealth (if applicable)** | Strongly disagree  1 | 2 | 3 | 4 | Strongly agree  5 |
| --- | --- | --- | --- | --- | --- |
| 1. I think that I would like to use this system frequently. |  |  |  |  |  |
| 2. I found the system unnecessarily complex. |  |  |  |  |  |
| 3. I thought the system was easy to use. |  |  |  |  |  |
| 4. I think that I would need the support of a technical person to be able to use this system. |  |  |  |  |  |
| 5. I found the various functions in this system were well integrated. |  |  |  |  |  |
| 6. I thought there was too much inconsistency in this system. |  |  |  |  |  |
| 7. I would imagine that most people would learn to use this system very quickly. |  |  |  |  |  |
| 8. I found the system very cumbersome to use. |  |  |  |  |  |
| 9. I felt very confident using the system. |  |  |  |  |  |
| 10. I needed to learn a lot of things before I could get going with this system. |  |  |  |  |  |

**Comparison: If you had your choice, would you want the evaluation to be done in person, by televideo, or do you not have a preference?** How has this consultation compare to others you may have had before? What parts were better, what parts were worse?

**E. How would you rank the following in terms of preference? 1 being the most preferred, 3 being the least preferred?**

| Evaluation in-person at the ED you presented to |  |
| --- | --- |
| Evaluation in-person at University ED in Worcester (involves a transfer) |  |
| Telehealth evaluation in the ED you presented to |  |

**Would you recommend that we offer future consultations by telehealth? Why, why not?**

1. *IMPLEMENTATION*

**How do you think we should prepare a patient for a telehealth consultation?** What information or instructions should we give them?

**How do you think we should support a patient during a telehealth consultation?** What support should be on hand for the patient? Anything we should do afterwards?

1. Wrap-up

Thank you so much for your time!
